# Describing and characterizing the *WAK/WAKL* gene family across plant species: a systematic review

**DOI:** 10.3389/fpls.2024.1467148

**Published:** 2024-11-12

**Authors:** Aaron Harvey, Noëlani van den Berg, Velushka Swart

**Affiliations:** Hans Merensky Chair in Avocado Research, Forestry & Agricultural Biotechnology Institute (FABI), Department of Biochemistry, Genetics and Microbiology (BGM), University of Pretoria, Pretoria, South Africa

**Keywords:** wall-associated kinase, wall-associated kinase-like, gene identification and classification, expression data, *cis*-acting elements

## Abstract

Wall-associated kinases (WAKs) and WAK-likes (WAKLs) are transmembrane pectin receptors which have seen rising interest in recent years due to their roles in stress responses and developmental pathways. Consequently, the genes encoding these proteins are continuously identified, described and characterised across a wide variety of plant species. The primary goal of characterizing these genes is to classify, describe and infer cellular function, mostly through *in silico* methods. However, inconsistencies across characterizations have led to discrepancies in WAK/WAKL definitions resulting in sequences being classified as a WAK in one study but as a WAKL or not identified in another. The methods of characterization range widely with different combinations of analyses being conducted, to similar analyses but with varying inputs and parameters which are impacting the outputs. This review collates current knowledge about *WAK/WAKL* genes and the recent characterizations of this family and suggests a more robust strategy for increased consistency among the different gene members, as well as the characterizations thereof.

## 1 Introduction

Wall-associated kinase (WAK) proteins are transmembrane pectin receptors that constitute one of the 15 receptor-like kinase (RLK) subfamilies and serve multiple functions within plant cells (Stephens et al., 2022). The first WAK protein (AtWAK1) was described in *Arabidopsis thaliana* using immunohistochemistry to confirm its association with the cell wall, and protease experiments to confirm the cytoplasmic kinase domain, thereby defining a protein capable of facilitating signaling between the cell wall and the cytoplasm (He et al., 1996). Subsequently, WAK-like (WAKL) proteins were also described in *A. thaliana* (Verica and He, 2002).

WAKs bind pectin in multiple forms, such as native pectin to influence cell expansion during plant development, and oligogalacturonides (fragmented pectin) that act as damage-associated molecular patterns (DAMPs) during abiotic and biotic stresses (Kohorn and Kohorn, 2012; Kohorn, 2015). Some WAK/WAKLs have been shown to confer resistance to hemibiotrophic and necrotrophic fungi through a range of mechanisms including pathogen- or host-derived elicitor detection and cell wall restructuring (Stephens et al., 2022).

The original *WAK/WAKL* family identified in *A. thaliana* consisted of five *AtWAKs* and 21 *AtWAKLs*. The *WAK/WAKL* gene families have since been characterised in many plant species, including *Solanum tuberosum* (potato), *Gossypium hirsutum* (cotton) and *Triticum aestivum* (bread wheat) which contained 29, 99 and 320 *WAK* and/or *WAKL* genes, respectively (Xia et al., 2022; Yu et al., 2022; Zhang et al., 2021).

Over the past two decades the *WAK/WAKL* gene family has been a subject of interest due to its involvement in plant defense and development (Stephens et al., 2022). The gene family has been bioinformatically and functionally characterised across several plant species to identify candidate genes involved in the successful defense response, highlighting their potential for improving crop disease resistance (Stephens et al., 2022). Recent characterizations of this gene family typically include the number of *WAK/WAKLs* identified within a species, predicted protein properties, phylogenetic analysis, gene structure visualization, and chromosome placement. The characterization studies also provide information on expression patterns in different tissues, under stress conditions, phytohormone introduction, the presence of conserved protein motifs and *cis-*acting elements in gene promoter regions, selection pressure predictions, subcellular localization and duplication predictions.

This review will synthesize and compare the *WAK/WAKL* gene families across plant species, the methodology used to identify, classify and characterize these genes, and propose improvements to increase consistency and comparability between different plant species and studies.

## 2 Identification of WAKs and WAKLs

### 2.1 Discovery of WAK/WAKLs and protein domain composition

AtWAK1 was first described as a transmembrane cell-wall associated protein with a cytoplasmic kinase domain (the serine/threonine protein kinase domain, pkinase) and extracellular epidermal growth factor (EGF)-containing regions (He et al., 1996). Subsequently, four additional WAKs were described (AtWAK2-5) sharing similar protein structure and domain compositions, including an N-terminal signal peptide (He et al., 1999). WAK proteins have been shown to be involved in cellular expansion and capable of binding to pectin within the plant cell wall. A homogalacturonan-binding region (later described as a GUB_WAK_bind domain, GWB) was identified near the N-terminal of AtWAK1, facilitating pectin binding (Decreux et al., 2006; Wagner and Kohorn, 2001). Using AtWAK1 as the query in a BLAST search, 22 proteins in *A. thaliana* were found to have high similarity to AtWAKs but lacked some functional domains and were consequently designated as AtWAKLs (Verica and He, 2002). Amongst these, five AtWAKLs were truncated proteins while the rest were transmembrane proteins containing a cytoplasmic kinase domain, an N-terminal signal peptide and extracellular EGF-like or calcium-binding EGF-like domains (some of which were degenerate). The truncated AtWAKLs lacked a transmembrane domain or the EGF-like domains; however, the expression of at least three of these *AtWAKL*s suggests that they may still be functionally important. The definition of a WAK and WAKL based on protein domain composition varies across recent studies, leading to discrepancies in classification.

### 2.2 Current approaches and inconsistency in the identification and classification of WAK/WAKLs

The main approach for identifying putative WAKs and WAKLs involves using the *A. thaliana* WAK/WAKL protein sequences as queries in a BLASTp (BLAST-protein) search against the proteome of the species of interest. Another method involves constructing a Hidden Markov Model (HMM) profile that includes sequences of the GWB domain-, EGF domain- and STK (or pkinase) domain, usually obtained from the Pfam database. Only candidate proteins (with their corresponding genes) containing both the kinase (STK) domain and the GWB domain are retained for further classification. The verification of protein identity and further classification of these proteins as either a WAK or WAKL are performed using protein domain prediction tools such as SMART, NCBI conserved domain search, and/or IntroPro (Pfam databases). However, inconsistencies in the recent identifications of WAK/WAKLs arise from varying queries, approaches and thresholds. For example, in *Juglans regia* (walnut), only AtWAKs were used in a local BLASTp (LBp) search, whereas in *Rosa chinensis* (rose) both AtWAKs and AtWAKLs were used in an LBp followed by an HMM search with three WAK-expected protein domains (**Table 1**). Furthermore, differing e-values during the identification of WAK/WAKLs impact the statistical confidence with which a gene is identified as a member of the WAK/WAKL family. For example, potential genes identified in rose with associated e-value between 1e-3 and 1e-5 might be retained in one study but excluded in another using stricter threshold values, such as those used in *Medicago truncatula* (barrel medic) *WAK/WAKL* identification (Kong et al., 2023; Liu et al., 2021).

**Table 1:** Methods and protein domains used for identification and classification of predicted WAK and WAKL proteins across various plant species.

Species (common name)	Identification approach (Query/e-value)	WAK vs. WAKL: Protein domains used for classification	Reference
*Brassica rapa* (Chinese cabbage)	LBp & LBn (AtWAK & AtWAKL/< 0.01)	WAK: WAK or GWB &/or WAKa & EGF-like/EGF_CA & pk & TM	Zhang et al. (2020)
		WAKL: pk &/or WAK/GWB or EGF	
*Cannabis sativa* (Cannabis)	LBp (AtWAK & AtWAKL/< 1e-5)	WAK: GWB & EGF/EGF_CA & pk & SP & TM	Sipahi et al. (2022)
		WAKL: pk &/or GWB &/or EGF/EGF_CA &/or SP &/or TM	
*Hordeum vulgare* (Barley)	HMM + TBLASTn (EGF_CA & pk & GWB**/**< -100)	N/A	Tripathi et al. (2021)
*Juglans mandshurica* (Walnut)	LBp (AtWAK/< 1e-5)	WAK: WAK/GWB & STK/PKc, EGF/EGF_CA & SP & TM	Li et al. (2022)
*Juglans regia* (Walnut)		WAKL: WAK/GWB & STK/PKc, EGF/EGF_CA &/or SP &/or TM	
*Malus domestica* (Apple)	Not specified (EGF & pk)	WAK: EGF & TM & pk	Zuo et al. (2019)
*Medicago truncatula* (Barrel medic)	BLASTp + HMM (AtWAK & OsWAK/< 1e-5)	WAK: EGF_CA & WAKa & GWB & pk_Tyr	Kong et al. (2023)
*Nicotiana benthamiana*	HMM (AtWAK & AtWAKL & SlWAK & SlWAKL)	WAK: GWB & EGF & pk	Zhong et al. (2023)
		WAKL: GWB &/or EGF &/or pk (Two of three)	
*Pisum sativum* (Pea)	LBp (AtWAK/< 1e-5)	WAK: STK/PKc_like & GWB	Li et al. (2023)
*Rosa chinensis* (Rose)	LBp (AtWAK & AtWAKL) + HMM (EGF_CA, GWB, pk_Ser-Thr/<1e-3)	WAK: EGF_CA, GWB, pk, SP, TM	Liu et al. (2021)
		WAKL: SP, TM, pk &/or EGF_CA and/or GWB	
*Saccharum spontaneum* (Wild sugarcane)	HMM (GWB & EGF & pk)	WAK: GWB & EGF & pk	Wang et al. (2023)
*Sorghum bicolor* (Sorghum)			
*Sesame indicum* (Sesame)	LBp (AtWAKLs/<10^-10^)	WAKL: GWB & pk	Yan et al. (2023)
*Solanum tuberosum* (Potato)	HMM	WAK: GUB_WAK/WAKa/EGF & TM & pk	Yu et al. (2022)
		WAKL: TM & pk &/or GUB_WAK/WAKa/EGF	
*Triticum aestivum* (Bread wheat)	LBp (AtWAK) + HMM (WAK family)	WAK: GWB & TM & pk	Xia et al. (2022)
*Zea mays* (Maize)	HMM (GWB & EGF_CA & pk/< 0.001)	WAKL: GWB & EGF_CA &/or pk	Hu et al. (2023)

**Figure 1:**
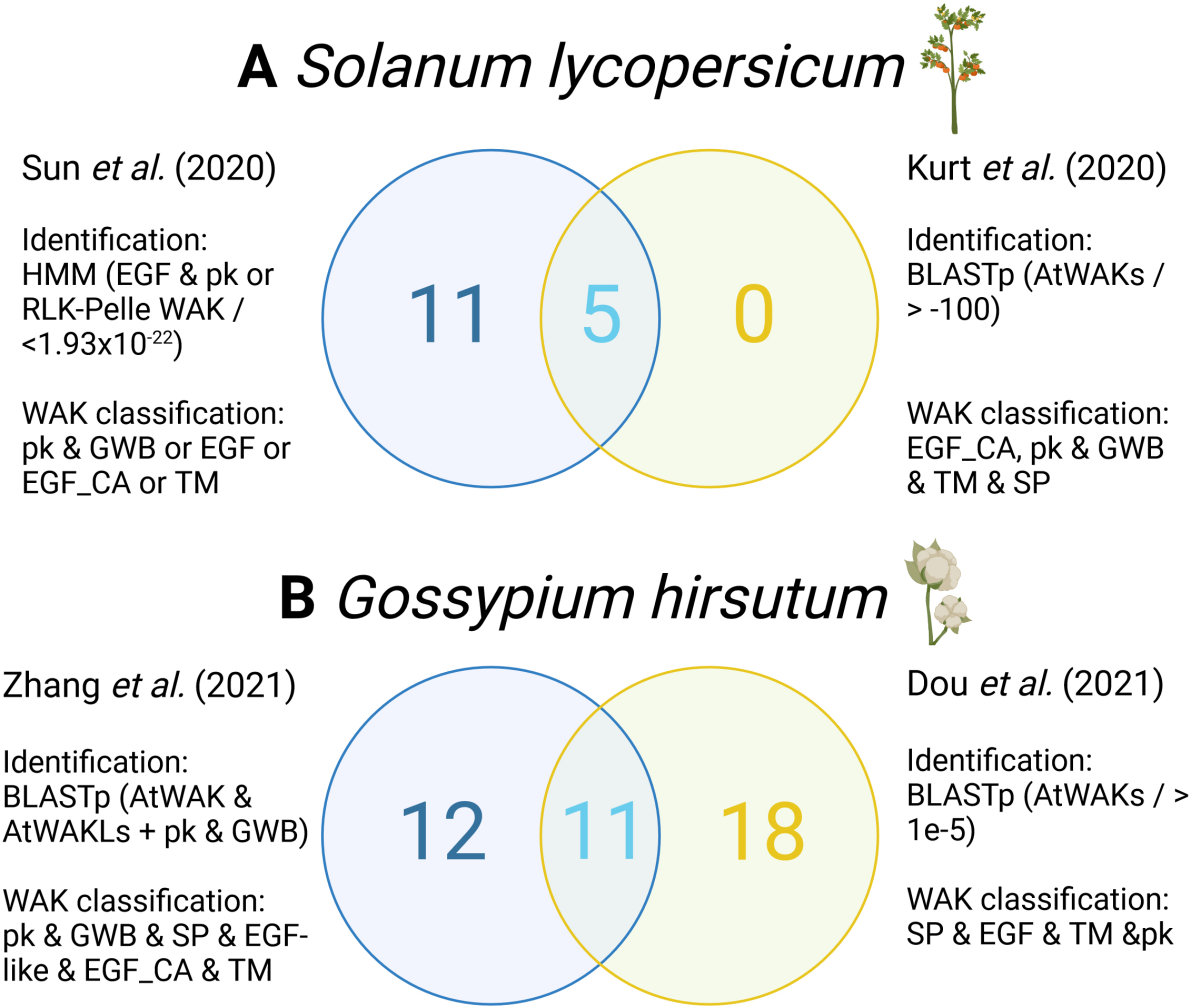
Comparison of WAK protein identification and classification methodology and the number and composition of WAK proteins in **(A)** *Solanum lycopersicum* (tomato) and **(B)** *Gossypium hirsutum* (cotton). (Created with BioRender.com).

*Gossypium hirsutum* (Cotton) and *Solanum lycopersicum* (Tomato) summarized in **Figure 1**.

HMM, Hidden Markov model search; LBp, Local BLASTp; LBn, Local BLASTn; pk, pkinase; GWB, GUB_WAK_bind; TM, Transmembrane; WAKa, WAK_assoc; STK, STKc_IRAK; PKc, PKc_Superfamily; SP, Signal peptide.

& = must include the previous and following domain.

Or = Must contain either the previous or following domain.

The criteria for protein domain architecture verification and classification of WAK/WAKLs also vary across different studies. In *Cannabis sativa* (cannabis), all five of the previously described protein domains associated with WAK proteins were used for classification, whereas other studies used different criteria (Sipahi et al., 2022). A study on *Malus domestica* (apple) only investigated the presence of an EGF, TM and pkinase domain to define their WAK proteins (Zuo et al., 2019). Differences in classification criteria typically involve the exclusion of the TM and signal peptide (SP) domains in WAKs. Among studies that classified WAKLs, the criteria also varied from a minimum of a pkinase or GWB domain in *Brassica rapa* (Chinese cabbage) to a minimum of a TM, SP and pkinase domain in rose (Liu et al., 2021; Zhang et al., 2020). Therefore, the WAKs identified in apple and Cannabis, as well as WAKLs from Chinese cabbage and rose are not comparable due to differing criteria, a predicament that applies to many recent characterizations (**Table 1**). Examples of this inconsistency is also evident in the WAK/WAKL characterization of *Solanum lycopersicum* (tomato) and *Gossypium hirsutum* (cotton) (**Figure 1**).

Inconsistencies in the methods used for WAK/WAKL identification has led to differences in the *WAK*/*WAKL* genes identified. For example, two independent characterizations of tomato WAKs yielded different results, with one identifying 16 and the other five WAKs, with only five being shared (**Figure 1A**; **Supplementary Table 1**). One difference was the use of different genomes, the *S. lycopersicum* L. SL4.0 genome from an open-source database (https://solgenomics.net/organism/Solanum_lycopersicum/genome/) and the genome from Phytozome V12.1 (https://phytozome.jgi.doe.gov/pz/portal.html - specific genome and annotation unspecified). The different genomes may have contributed minorly, but a more likely explanation is the use of different identification methods (with different e-value cutoffs) and classification protocols.

The number of cotton WAKs varied considerably between studies, with 11 shared and 12 and 18 unique to each of the respective studies (**Figure 1B**). Both studies sourced the cotton genome data from the same database (CottonGen - https://www.cottongen.org) with Dou et al. (2021) using the TM-1 genome ZJU_v2.1. and Zhang et al. (2021) likely (genome not specified) using the TM-1 genome UTX_v2.1. Despite both studies using BLASTp for WAK identification they employed different inputs and domains for classification, with one study including a GWB and an additional EGF_CA domain. Even when the identification processes are similar, differences in criteria lead to varying repertoires of WAKs being identified.

**Figure 2:**
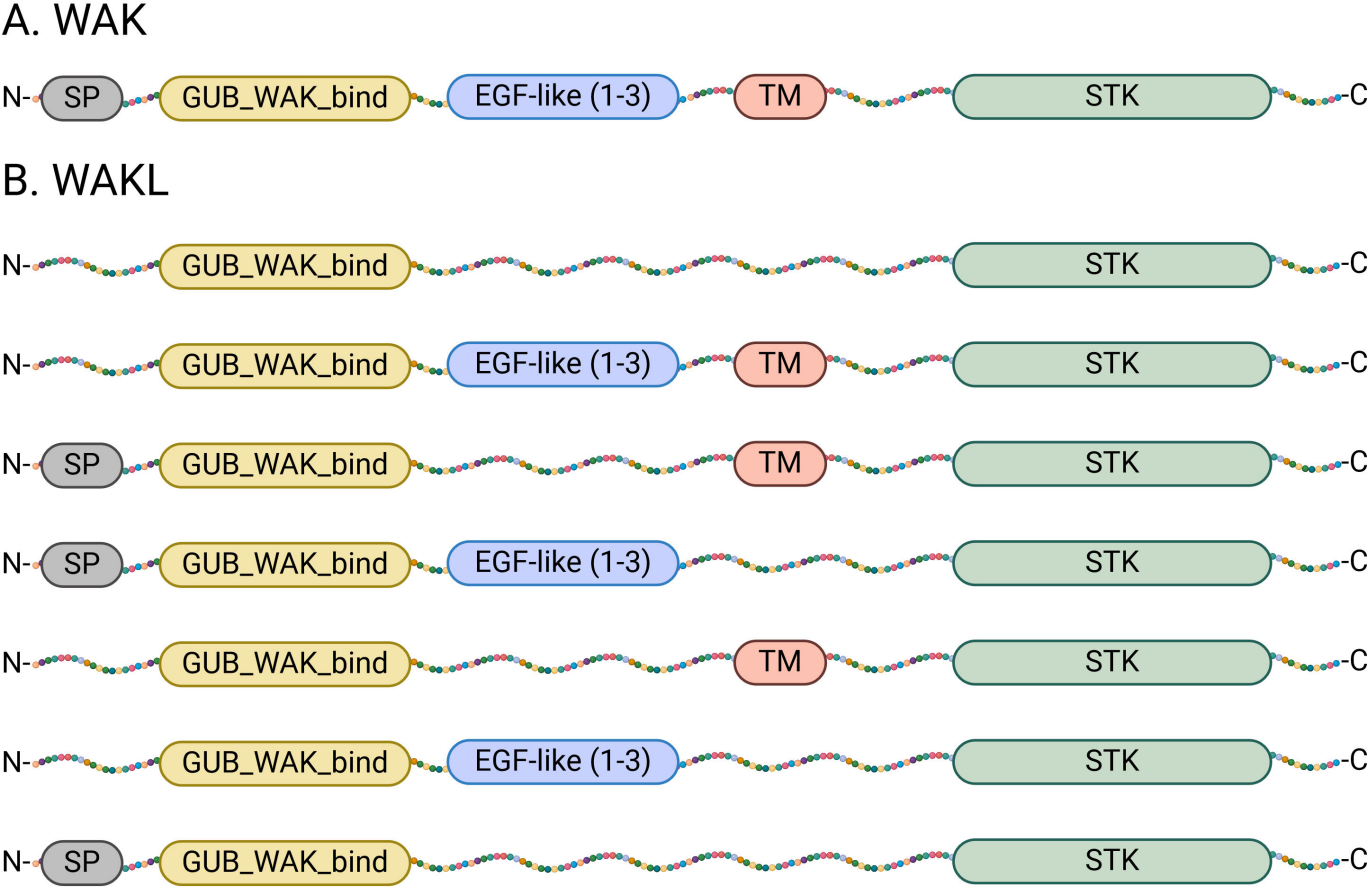
Model of required protein domains for the classification of WAK and WAKL proteins. **(A)** Model of the protein domain architecture of WAK proteins. The five domains required are a signal peptide (SP) at the N-terminal (grey), a galacturonan-binding domain (GUB_WAK_Bind; yellow), one to three extracellular epidermal growth factor (EGF)-like domains or EGF_Ca (blue), a transmembrane (TM) domain (Red) and a cytoplasmic Ser/Thr kinase (STK) domain (also known as a pkinase domain at the C-terminal; green). **(B)** Model of the different combinations of protein domains that make up WAKL proteins. They must contain an STK domain and a GUB_WAK_bind domain, but the other domains are variable. (Created with BioRender.com).

To reduce inconsistencies and enable better comparison of this gene family across plant species, future WAK/WAKL characterizations should follow consistent strategies. While identification methods can vary (using BLASTp or HMM searches) the verification and classification process should have the same backbone (such as the inclusion of at least the AtWAK/WAKLs protein sequences as the input query) to minimize the possibility of missed gene members. The verification and classification should however maintain high levels of consistency. Based on the original discovery and descriptions of AtWAK and AtWAKLs, the following model proposes a way to increase standardization: WAK proteins must have an SP, GWB, EGF-like, TM and STK domain in tandem, whereas WAKLs must contain at least a GWB and STK domains with possible varying combinations of the SP, EGF-like and TM domains (**Figure 2**).

## 3 *WAK* and *WAKL* composition of species recently characterised

It is important to distinguish between WAK and WAKLs as they could have similar functions, but in different locations in the cell, influencing protein function and response to stimuli. WAKs are defined as transmembrane pectin receptors whereas WAKLs (which are not all transmembrane proteins) should not be classified under the same group. Misclassification could lead to unreliable conclusions as not all members will conform to the same definition. Between 5 and 320 *WAKs* have been identified across various plant species (**Table 2**). The number of WAKs positively correlates with genome size, but not with ploidy or plant type (monocotyledon or dicotyledon). Bread wheat, a hexaploid, has 320 *WAKs* which is significantly higher than other species (**Table 2**) (Xia et al., 2022). This could be due to its hexaploid nature, however, this trend is not consistent across other species. For example, the tetraploid potato has fewer *WAKs* (16) compared to cannabis (23), a diploid (Sipahi et al., 2022; Yu et al., 2022). More research is needed to confirm the correlation between ploidy and the number of *WAK/WAKL* genes. Generally, higher ploidy species tend to have more copies of protein-coding genes but the larger number of *WAKs* could be due to the criteria used for classification. In bread wheat some TaWAKs, such as TraesCS3A02G033400, (and others in phylogenetic group 3), lack an EGF domain and others, like TraesCS7D02G085600, lack an SP which would classify them as WAKLs under the proposed model, thereby reducing the total number of TaWAKs. The less stringent criteria used in the bread wheat study may have contributed to the higher number of TaWAKs identified (Xia et al., 2022).

**Table 2:** Summary of the number of *WAK*s and *WAKL*s identified in various plant species.

Species (Common name)	Ploidy	Genome size (Mb)	Monocot or Dicot	Number of WAKs	Number of WAKLs	Reference
*Brassica rapa* (Chinese cabbage)	Allotetraploid	352.8	Dicot	11	85	Zhang et al. (2020)
*Cannabis sativa* (Cannabis)	Diploid	875.7	Dicot	23	30	Sipahi et al. (2022)
*Gossypium arboreum* (Cotton)	Diploid	1 621	Dicot	16	42	Zhang et al. (2021)
*Gossypium raimondii* (Cotton)	Diploid	750.2	Dicot	11	55	
*Gossypium hirsutum* (Cotton)	Allotetraploid	~2 250-2 430	Dicot	23	76	
*Gossypium hirsutum* (Cotton)	Allotetraploid	~2 250-2 430	Dicot	29	N/A	Dou et al. (2021)
*Hordeum vulgare* (Barley)	Diploid	4 226	Monocot	91	N/A	Tripathi et al. (2021)
*Juglans mandshurica* (Walnut)	Diploid	528.2	Dicot	5	9	Li et al. (2022)
*Juglans regia* (Walnut)	Diploid	572.8	Dicot	11	16	
*Malus domestica* (Apple)	Di- or triploid	703	Dicot	44	N/A	Zuo et al. (2019)
*Medicago truncatula* (Barrel medic)	Diploid	429.6	Dicot	54	N/A	Kong et al. (2023)
*Nicotiana benthamiana* (Tobacco)	Amphidiploid	N/A	Dicot	15	23	Zhong et al. (2023)
*Pisum sativum* (Pea)	Diploid	3 796	Dicot	24	N/A	Li et al. (2023)
*Rosa chinensis* (Rose)	Diploid	515.1	Dicot	23	45	Liu et al. (2021)
*Saccharum spontaneum* (Wild sugarcane)	Autopolyploid	2 761	Monocot	19	N/A	Wang et al. (2023)
*Sorghum bicolor* (Sorghum)	Diploid	708.8	Monocot	37	N/A	
*Sesame indicum* (Sesame)	Diploid	357	Dicot	N/A	31	Yan et al. (2023)
*Solanum lycopersicum* (Tomato)	Diploid	950	Dicot	11	18	Sun et al. (2020)
*Solanum lycopersicum* (Tomato)	Diploid	950	Dicot	5	N/A	Kurt et al. (2020)
*Solanum tuberosum* (Potato)	Tetraploid	705.8	Dicot	16	13	Yu et al. (2022)
*Triticum aestivum* (bread wheat)	Hexaploid	14 567	Monocot	320	N/A	Xia et al. (2022)
*Zea mays* (Maize)	Diploid	2 400	Monocot	N/A	58	Hu et al. (2023)

N/A means that that particular data was not discussed in the relevant publication.

The number of *WAKL* genes in plant species ranges from 9-82, generally being in higher abundance than the *WAKs* in most species with potato being an exception (**Table 2**). This could be due to fewer domains required for WAKL protein classification, as fewer domains in coexistence increase the probability of occurrence. This could also result from duplication events of *WAKs*, leading to neofunctionalism, where some domain-coding regions mutate after duplication, allowing for new adaptive functions (Roulin et al., 2013). The exception in potato (16 *StWAKs* vs 13 *StWAKLs*) might be due to different criteria used for StWAKL classification (which lack a TM-domain) unlike other studies. This suggests that potato WAKLs are likely membrane-bound proteins, but other characterised WAKLs lacking a TM domain could be functionally important. Therefore, important potato WAKLs, without TM domains, involved in functions such as pathogen defense might be unidentified and uncharacterised. Not all studies have characterised *WAKLs* in the species studied, thus the data set for *WAKLs* is incomplete. However, *WAKLs* have been shown to play important roles in plants, with AtWAKL10 and OsWAKL21.2 involved in resistance to *Pseudomonas syringae* and *Xanthomonas oryzae*, respectively. This illustrates the value of identifying WAKLs for potential further analyses (Bot et al., 2019; Malukani et al., 2020).

## 4 Gene comparisons

### 4.1 *WAK/WAKL* gene structure, placement and duplication methods

The *WAK/WAKL* gene family shows variation in gene structure, with the number of exons ranging from 1-27 exons per gene (**Table 3**). Some conservation of exon-intron structure (gene structure) exists between monocots and dicots, with most immunity-related WAKs (across plant species) typically having three or four exons, with the first exon being the largest (Stephens et al., 2022). The average number of exons in this family ranges from three to five with open reading frames (ORF) that span between 0.8-330 kb. However, the lack of correlation between ORF length and exon number with gene function (excluding immunity-related WAKs) discounts these properties as a predictive marker for a gene’s role. Identifying tandemly duplicated pairs and gene placement is essential for evolutionary analyses to understand the history and potential future of this gene family.

**Table 3:** *WAK* and *WAKL* gene information from various species.

Species	Chromosome (chr) placement	Number of exons	Mode number of exons	ORF (Kb)	Clusters ^a^ (on chr_)	Tandem duplications predicted (%)	K_a_/K_s_ values	Reference
*Brassica rapa*	On 10/10 chrs	1-27	>3	0.8-20	1, 2, 5, 6, 7, 8	55.17	N/A	Zhang et al. (2020)
*Cannabis sativa*	On 6/10 chrs	2-7	3	2.5-15	1,4,7	8.7	All < 1 One > 1	Sipahi et al. (2022)
*Gossypium arboreum*	On 11/13 chrs	N/A	N/A	N/A	N/A	N/A	N/A	Zhang et al. (2021)
*Gossypium raimondii*	On 11/13 chrs	N/A	N/A	N/A	N/A	N/A	N/A	
*Gossypium hirsutum*	On 19/23 chrs	1-8	3	2-18	A2, A3, A5, A9, A10, A11, D2, D5, D9, D10, D11	15.15	All < 1	
*Gossypium hirsutum*	On 12/26 chrs	1-8	3	1-12	A2, A5, D2, D10	17.24	All < 1	Dou et al. (2021)
*Juglans mandshurica*	On 7/16 chrs	2-8	5	4-330	15	64.29	Most < 1 Two pairs > 1	Li et al. (2022)
*Juglans regia*	On 8/16 chrs		4		6,10	70.37		
*Malus domestica*	On 16/17 chrs	1-11	3/4	1.5-5	N/A	N/A	N/A	Zuo et al. (2019)
*Medicago truncatula*	On 8/8 chrs	2-4	3	2.8-6.5	1,3	18.52	N/A	Kong et al. (2023)
*Nicotiana benthamiana*	N/A	2-8	>2	1.3-13	N/A	N/A	N/A	Zhong et al. (2023)
*Pisum sativum*	On 7/7 chrs	1-	>3	2-27	1,3,6	10.71	N/A	Li et al. (2023)
*Rosa chinensis*	On 7/7 chrs	1-12	3/4	1.8-15.5	1, 2, 5, 7	N/A	N/A	Liu et al. (2021)
*Saccharum spontaneum*	On 21/32 chrs	1-8	3/4	2.5-25	None	11	All < 1	Wang et al. (2023)
*Sorghum bicolor*	On 9/10 chrs				4, 7	28		
*Sesame indicum*	On 8/13 chrs	2-5	3	2-23	3, 5, 6, 8, 10, 11, 12	51.6	N/A	Yan et al. (2023)
*Solanum lycopersicum*	On 9/12 chrs	1-6	2-3	1-6.5	5, 9, 10	N/A	N/A	Sun et al. (2020)
*Solanum lycopersicum*	On 2/12 chrs	3-4	3	N/A	7, 9	N/A	N/A	Kurt et al. (2020)
*Solanum tuberosum*	On 8/24 chrs	1-10	3/4	1.9-10.2	5, 9, 10	72.41	All < 1	Yu et al. (2022)
*Zea mays*	On 10/10 chrs	2-5	3	3-19.5	1,2,3,6,8	16	All < 1	Hu et al. (2023)

a A cluster refers to two or more genes within a few thousand base pairs of each other.

N/A means that that particular data was not discussed in the relevant publication.

ORF, number of exons, mode of exons and clustering estimated off gene structure visualizations when not stated in-text.

A noticeable feature of the chromosomal placement of these genes is their clustering across multiple chromosomes in species such as Chinese cabbage, rose and *Sesamum indicum* (sesame) (**Table 3**). This can be indicative of tandem duplication events occurring, which in turn allows for the expansion of this gene family within a species (Kong et al., 2007). Duplication events involve the replication and insertion of DNA segments into a location close or far away from the original DNA section, producing two copies of a DNA segment. The main duplication mechanisms predicted in the characterization of the *WAK/WAKL* gene families through *in silico* analyses include whole-genome duplications, tandem duplication, proximal duplication and segmental duplication (Lallemand et al., 2020). Tandem duplications were the major type of duplication in Chinese cabbage, walnut, and potato, while, whole-genome-, segmental- and proximal duplications also contributed to *WAK* gene expansion in cotton, Chinese cabbage, *Saccharum spontaneum*, a *Saccharum* hybrid and *Sorghum bicolor* (wild sugarcane and sorghum) (Dou et al., 2021; Wang et al., 2023; Zhang et al., 2020). The type and frequency of duplication events likely depend on the genome organization (ploidy), evolutionary history (recent or distant speciation or hybridization), and current environmental influences (adaptation processes).

For *WAK/WAKL* gene pairs originating from tandem duplication events, nonsynonymous-to-synonymous (K_a_/K_s_) ratios were calculated to predict the type of evolutionary pressure acting on these pairs. Nonsynonymous mutations (K_a_) represent mutations resulting in amino acid changes, while synonymous mutations (K_s_) represent mutations resulting in the same amino acid. The K_a_/K_s_ test is an empirical method to predict the type of selection influencing gene evolution, although this method is best suited towards long, single exon genes with relatively low false-positive and false-negative rates compared to other methods (Nekrutenko et al., 2002). Most gene pairs in **Table 3** have K_a_/K_s_ ratios below one (<1), indicative of purifying selection, where deleterious polymorphisms are removed, reducing genetic diversity (Cvijović et al., 2018). This purifying selection likely removes truncated, non-functional proteins while preserving their function. However, one *WAK/WAKL* pair in cannabis and two in walnut show ratios greater than one (>1), indicating positive selection, where advantageous mutations are selected for and become fixed over time (Li et al., 2022; Sipahi et al., 2022; Thiltgen et al., 2017). The hypothesis for the walnut WAK/WAKL pairs under positive selection suggests that variation in the specific duplicated genes, increases the rate of neofunctionalism, allowing for novel or contributory functions to arise (Li et al., 2022). The WAK/WAKLs encoded by these genes may enhance resistance to a specific- or several- or new pathogens or provide developmental advantages.

### 4.2 *Cis*-acting element comparison in *WAK/WAKL* promotor regions

**Figure 3:**
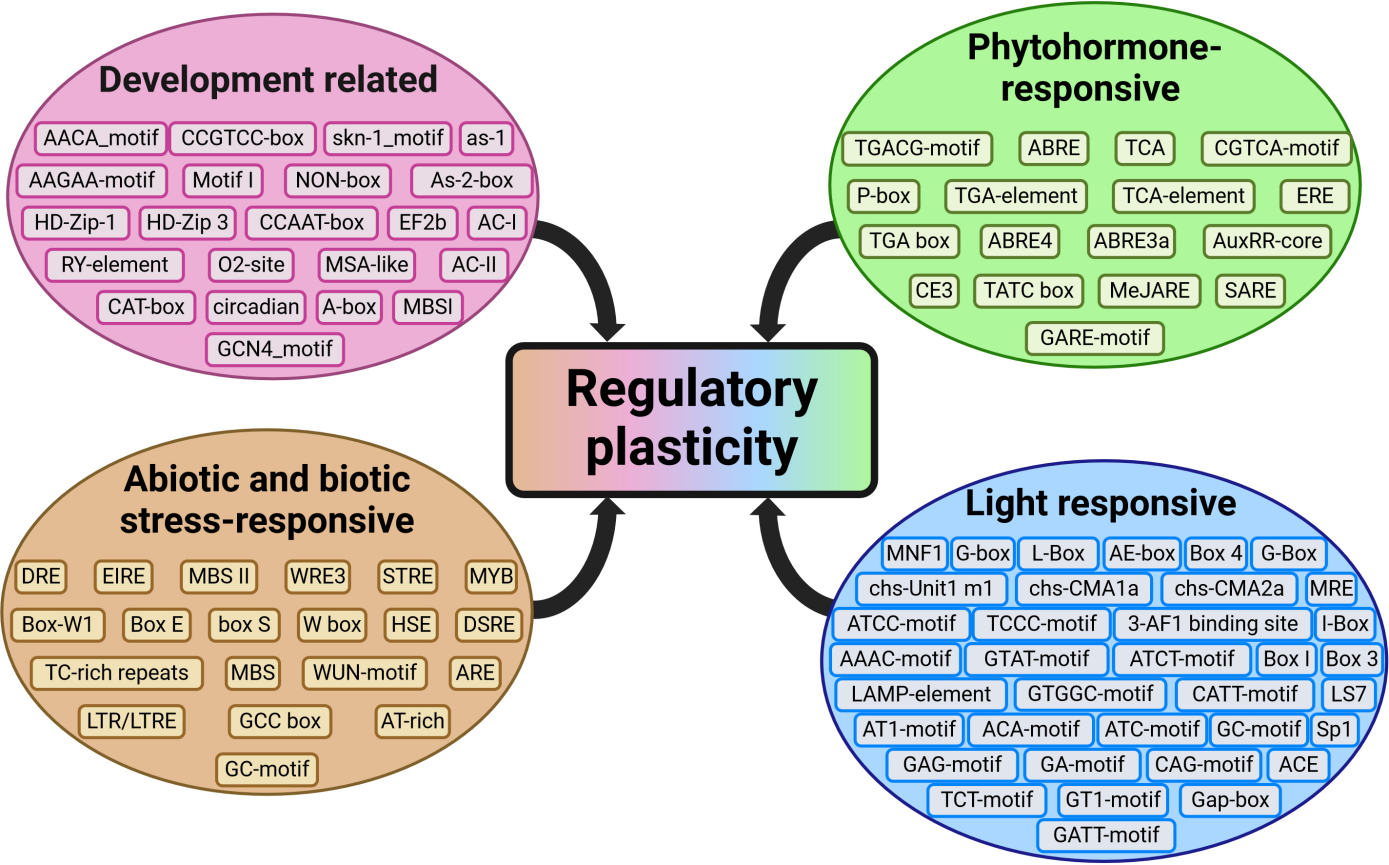
*Cis*-acting elements predicted in the promoter regions (which varied between 1000 and 2000 base pairs upstream of the start site) of *WAK/WAKL* gene families, which contribute to regulatory plasticity. The elements were predicted in walnut, cotton, apple, cannabis, potato, tomato, barley, barrel medic, *Nicotiana benthamiana*, wild sugarcane, and sorghum (Dou et al., 2021; Kong et al., 2023; Li et al., 2022; Sipahi et al., 2022; Tripathi et al., 2021; Wang et al., 2023; Yu et al., 2022; Zhang et al., 2021; Zhong et al., 2023; Zuo et al., 2019). (Created with BioRender.com).

*Cis*-acting elements in the promoter regions of genes significantly contribute to differential regulation (Biłas et al., 2016). These elements involved in phytohormone-responsive, light-responsive, biotic- and abiotic-stress responsive, and development-related pathways, enhance regulatory plasticity, allowing genes to be selectively induced or suppressed under certain conditions to perform specific roles (**Figure 3**).

Elements responsive to developmental pathways are present in the promoter regions of *WAK/WAKLs*, playing roles in leaf cell expansion during developmental stages and potentially in cell tension (Wagner and Kohorn, 2001). Predicted development-related *cis-*acting elements include the AAGAA-motif (endosperm-specific negative expression), AC (xylem-specific expression), O2-site (metabolism of the storage protein, zein) and CAT-box (meristem expression). For example, *CsWAK3* in cannabis contains a root-specific expression element, while *CsWAKL11* contains a seed-specific element, suggesting development-related tissue-specific regulation of these two genes (Sipahi et al., 2022).

Phytohormone-responsive c*is*-acting elements are also present in the promoter regions of many *WAK/WAKLs*, with some genes showing induction following phytohormone treatment. Phytohormones influence plant physiology including growth, development, and abiotic- and biotic stress responses (El Sabagh et al., 2022). Treatment with gibberellin and abscisic acid in potato and cotton induced five *WAKs* and 13 *WAKLs*, potentially due to the presence of ABRE (abscisic acid responsive element), P-box and TATC-box (gibberellin responsive) *cis-*acting elements (Dou et al., 2021; Yu et al., 2022). Other predicted phytohormone-responsive elements in *WAK/WAKL* promoters include those responsive to salicylic acid (TCA-element), methyl jasmonate (TGACG motif), auxin (TGA-box) and ethylene (ERE) in various species such as cotton, apple, potato, tomato and *Nicotiana benthamiana* (Sun et al., 2020; Zhang et al., 2021; Zhong et al., 2023; Zuo et al., 2019). Phytohormones play important roles across many cellular processes and understanding their involvement in these pathways can enhance our knowledge of their function.

Abiotic and biotic stress-responsive elements are implicated in mechanical wounding and defense response pathways in species such as bread wheat, walnut, rose, apple, potato, barrel medic, Chinese cabbage*, N. benthamiana*, and wild sugarcane (Kong et al., 2023; Li et al., 2022; Wang et al., 2023; Xia et al., 2022; Yu et al., 2022; Zhong et al., 2023; Zuo et al., 2019). Examples include the abiotic responsive element, LTR (low-temperature responsive), which regulates gene expression in colder climates and the biotic responsive element, W-box responsive to wounding and pathogen response. Approximately half of the identified tomato *WAK/WAKLs* (14 out of 29) contained the WUN-motif, with additional phytohormone responsive elements (methyl jasmonate, abscisic acid, gibberellin, salicylic acid and, auxin) in the promoter regions. The authors hypothesized that post-wounding rapid induction of *SlWAK-RLKs* may be mediated by these phytohormone pathways, although functional studies are needed (Sun et al., 2020).

In cotton, 13 and 10 *WAK/WAKLs* were induced by gibberellin and auxin, respectively (Dou et al., 2021). Putative gibberellin- and auxin-responsive elements complemented RT-qPCR expression data, linking these *cis*-acting elements directly to gene expression. In potato, gibberellin and auxin induced two and three *WAK/WAKLs*, respectively, with predicted *cis*-acting elements aligning with expression results (Yu et al., 2022). This provides evidence of *WAK/*WAKL involvement in phytohormone pathways and illustrates the relevance of *cis-*acting element predictions. These predictions can guide *in silico* studies regarding *WAK/WAKL* expression patterns in response to phytohormones before conducting expression work, potentially allowing for the editing or selection of promoter regions to control gene expression for specific developmental or defense pathways to maximize efficiency.

Light-responsive elements were predicted in apple, tomato and walnut, with common elements such as Box 4 and the TCT-motif (Li et al., 2022; Sun et al., 2020; Zuo et al., 2019). These elements may facilitate a ‘circadian clock’ of *WAK/WAKL* genes, as seen in other receptor-like kinases (Zuo et al., 2019). This suggests a time-specific induction or suppression of certain *WAK/WAKLs* to support time-sensitive cellular functions, potentially impacting growth patterns and allowing cellular expansion towards the light during the day.

While the results from these studies provide a foundation for comparison, discrepancies in prediction methods may impact general conclusions. Typically, *cis*-acting elements are predicted in the region 2000 bp upstream of a gene’s transcriptional start site, but this was not followed for apple and tomato, where 1500 bp promoters were used (Sun et al., 2020; Zuo et al., 2019). Reduced promoter size impacts the abundance of predicted elements, making comparisons difficult. Consistency in promoter length within the same gene family is crucial for comparability. The lack of e-value cutoffs for element prediction further complicates the comparison. Standardizing prediction methods would improve the comparability of this gene family across species, enabling larger-scale conclusions and more accurate analyses. Identifying common elements across species could provide research targets for studying gene regulation and function, potentially leading to overexpression or suppression of genes of interest in future studies or genetically modified organisms (GMOs) for desired outcomes.

## 5 Expression data comparison

Inferences about functional roles are made by evaluating gene expression patterns (here focusing on *WAK/WAKL*s). These studies can indicate the involvement of genes in specific tissues, phytohormone responses, abiotic stresses (such as drought and cold), and biotic stresses (such as pathogen infection). Upregulation during environmental changes is a primary line of evidence for implicating genes in particular responses. For example, cotton *WAK/WAKLs* were upregulated in response to cold, heat, salt, and chemically-induced drought stress suggesting their involvement, while upregulated barrel medic *WAK/WAKLs* were involved in the defense responses against *Macrophomina phaseolina* and *Ralstonia solanacearum* (Kong et al., 2023; Zhang et al., 2021). Expression data are typically obtained from RT-qPCR experiments or RNA-sequencing data, which is often validated with RT-qPCR. Unlike previous sections, expression analysis approaches were more consistent across various studies, increasing the reliability of the conclusions drawn.

### 5.1 Tissue specificity and developmental stages

Phylogenetic group-specific tissue expression was observed when assessing the Chinese cabbage *BrWAK/WAKLs*, with three genes expressed in roots, five in flowers and three in calluses (Zhang et al., 2020). This suggests a close correlation between *BrWAK/WAKL* evolutionary relationships and tissue specificity. Tissue-specific expression was assessed in cannabis, sesame, *N. benthamiana*, walnut and bread wheat in tissues such as roots, stems, leaves, pericarp, grains, spikes, buds, pods, seeds and flowers (Kong et al., 2023; Li et al., 2022; Sipahi et al., 2022; Xia et al., 2022; Yan et al., 2023; Zhong et al., 2023). In barrel medic, some *MtWAKs*, like *MtWAK24* and *MtWAK50* showed expression specifically in roots, while others, such as *MtWAK7* and *MtWAK8* were expressed in multiple tissues (Kong et al., 2023). These results indicate that *WAK/WAKL* genes can be expressed in a few or many different plant tissues simultaneously or exhibit tissue-specific expression.

Expression data during different developmental stages was obtained for cotton, showing the involvement of a subset of *WAK/WAKLs* in the initial developmental stages of fiber development, and another subset in the cellular elongation phase (Dou et al., 2021). In tomato, expression data for *WAK/WAKL*s shows the involvement of a subset of *SlWAK/SlWAKL*s in fruit development, with varying expression patterns during fruit expansion and ripening (Sun et al., 2020). Identifying and validating genes involved in functions such as ripening could aid in the selection of varieties during screening and breeding programs to produce larger fruits with controlled ripening times. Most of the barrel medic’s *MtWAKs* were downregulated during nodulation, except for the induction of *MtWAK1*, suggesting its contribution to the process (Kong et al., 2023). The *WAK/WAKL* repertoire can thus play a role in many different developmental stages and illustrates the specificity of function some *WAK/WAKL*s have.

### 5.2 Abiotic and biotic responses

When characterizing the *WAK/WAKL* gene family, one major focus is on linking gene subsets with abiotic and biotic stress responses. In cotton, expression data showed that *GhWAK/WAKLs* responded to abiotic factors including cold, heat, salt, and polyethylene glycol (drought simulation). Specifically, *GhWAK9* was induced in all four abiotic responses, while *GhWAKL17* was only involved in the cold response (Zhang et al., 2021). This demonstrates that *WAK/WAKL*s can have a universal or specific role during stress. Similarly, in Chinese cabbage, expression patterns during drought, high temperature, and high humidity stress showed an association of *BrWAK/WAKLs* in these abiotic responses (Zhang et al., 2020). In tomato, nine *WAK/WAKL*s were upregulated and three downregulated in response to mechanical wounding, indicating their role in the wounding response (Sun et al., 2020). In barrel medic, 10 *MtWAK*s were upregulated due to drought, seven due to cold and three due to salt stress, suggesting that a combined 12 *MtWAK*s are involved in multiple abiotic stress responses (Kong et al., 2023). These findings illustrate that WAK/WAKLs play significant roles in various abiotic stress responses, with specific genes involved in specific responses.

A subset of *WAK/WAKL*s have been associated with biotic defense responses through gene expression analyses, with some functionally shown to play a role in defense. *AtWAKL22* and *TaWAK2* are known to protect against *Fusarium oxysporum* and *Fusarium graminearum*, respectively (Diener and Ausubel, 2005; Gadaleta et al., 2019; Liu et al., 2021; Zhong et al., 2023). Upregulation during defense responses implicated 20 *WAK/WAKLs* in bread wheat, eight in walnut, 12 in rose, eight in sesame, and six in potato (Li et al., 2022; Liu et al., 2021; Xia et al., 2022; Yan et al., 2023; Yu et al., 2022). In barrel medic, nine out of 12 *MtWAK*s were induced by yeast elicitor treatment, one by *M. phaseolina* (necrotrophic fungus) infection, three by *R. solanacearum* (bacteria) infection and 10 by *Erysiphe pisi* (biotrophic fungus) (Kong et al., 2023). Different *WAK/WAKLs* were upregulated following *M. phaseolina* and *R. solanacearum* infection, suggesting pathogen-specific roles in the respective defense responses. Overexpression of *MtWAK24* in *N. benthamiana* leaves reduced lesion size following *Phytophthora parasitica* infection, validating its important role in the inhibition of *P. parasitica* infection (Kong et al., 2023).

Some studies initially used *in silico* expression data to suggest a gene’s involvement in defense and then confirmed some genes with functional work, thus validating the *in silico* predictions. Virus induced gene silencing (VIGS) was used in rose and *N. benthamiana* to silence *WAK/WAKLs* involved in defense, which increased disease severity. Silencing *RcWAK4* in rose enhanced susceptibility to *Botrytis cinerea* while silencing *NbWAK12* and *14* and *NbWAKL6* and *12* in *N. benthamina* increased susceptibility to tomato yellow curl leaf virus (Liu et al., 2021; Zhong et al., 2023). This molecular evidence substantiates the defense role of the selected *WAK/WAKLs*. While *in silico* predictions are the first step towards identifying potential defense-related genes, molecular validation (such as VIGS or other functional work) is essential to verify these predictions, ensuring accurate identification of *WAK/WAKLs* involved in stress responses before utilizing them in crop improvement.

## 6 Protein comparisons

### 6.1 General properties and subcellular localization

**Figure 4:**
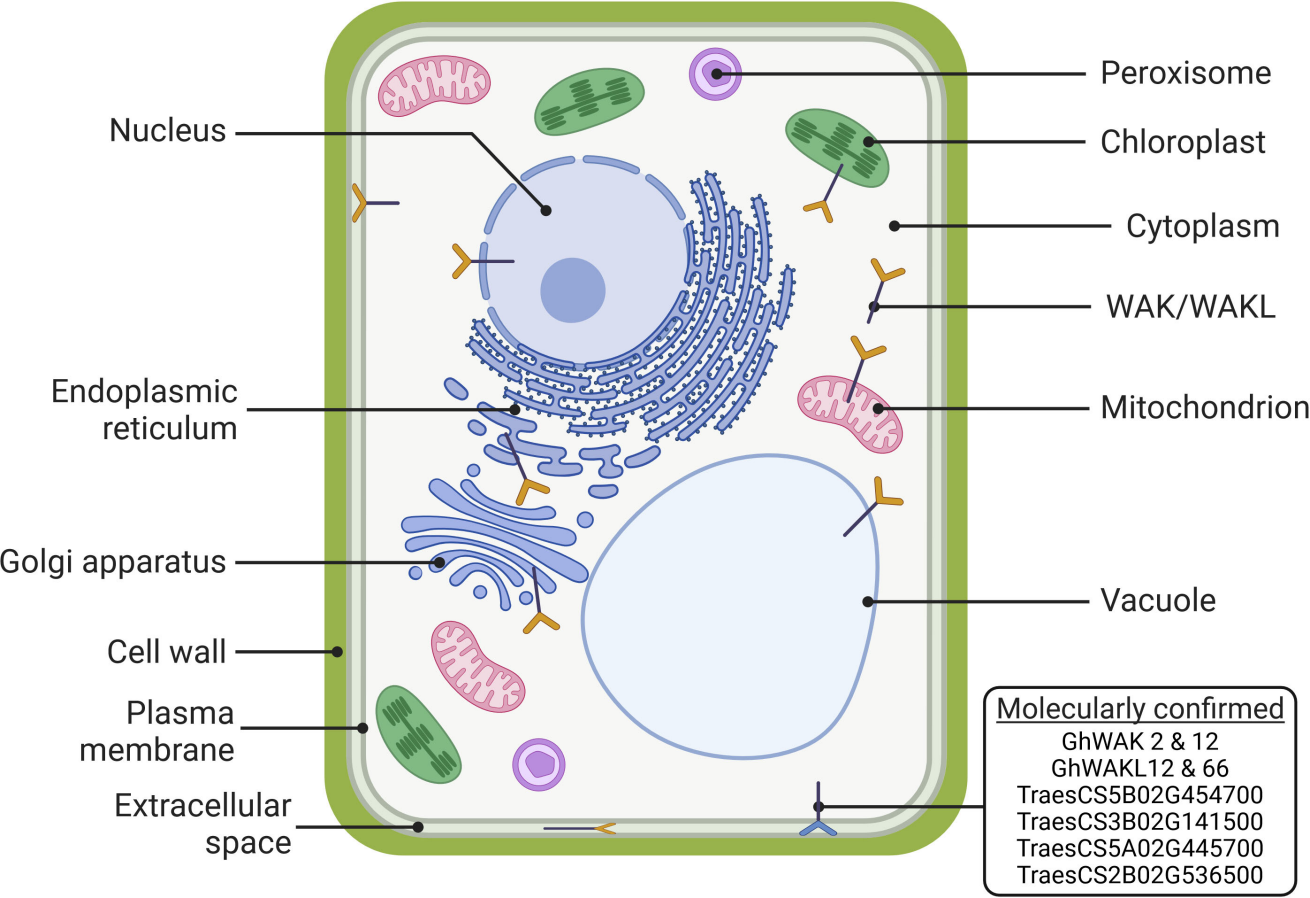
Subcellular localization predicted by *in silico* methods (yellow icons) or demonstrated through localization experiments (blue icon) of recently characterised WAK and WAKL proteins in bread wheat, walnut, cotton, apple, cannabis, pea, barrel medic, and potato (Kong et al., 2023; Li et al., 2022, 2023; Sipahi et al., 2022; Xia et al., 2022; Yu et al., 2022; Zhang et al., 2021; Zuo et al., 2019). (Created with BioRender.com).

The recently characterised WAK/WAKL proteins across species range between 200-3350 amino acids in length, 22.3-155.59 kDa in weight and 4.86-9.4 isoelectric points (**Table 4**). These proteins are larger with properties in the normal ranges for plant proteins (Mohanta et al., 2019; Ramírez-Sánchez et al., 2016). WAK proteins likely localize to the plasma membrane due to the presence of a transmembrane domain. However, WAKL proteins do not always contain a transmembrane domain, resulting in variable cellular localizations. *In silico* characterization of the WAK/WAKL family has predicted localizations to various other cellular structures, including the nucleus, chloroplast, extracellular matrix, mitochondria, endoplasmic reticulum, golgi body, vacuoles and the cytoplasm. Some of these localizations have been confirmed through experiments utilizing green fluorescent protein (GFP) markers (**Figure 4**), indicating that the WAK/WAKLs (specifically the WAKLs) can function throughout the cell, not just at the plasma membrane.

**Table 4:** WAK and WAKL proteins characterised between the year 2018-2023.

Species	WAK/WAKL protein length (aa)	WAK/WAKL protein weight (kDa)	WAK/WAKL protein pI	Reference
*Brassica rapa*	~200-3350	N/A	N/A	Zhang et al. (2020)
*Cannabis sativa*	582-983	65,6-108,8	5,80-8,96	Sipahi et al. (2022)
*Gossypium arboreum*	302-1049	48,86-117,73	5,00-9,20	Zhang et al. (2021)
*Gossypium raimondii*				
*Gossypium hirsutum*				
*Gossypium hirsutum*	606-1200	67.36-134.02	5.11-8.79	Dou et al. (2021)
*Juglans mandshurica*	629-1396	69.19-155.95	4,86-8,83	Li et al. (2022)
*Juglans regia*	513-839	57.57-92.13		
*Malus domestica*	302-998	33,63-110,53	5,1-9,26	Zuo et al. (2019)
*Medicago truncatula*	52-771	5.55-85.6	4.47-10.17	Kong et al. (2023)
*Nicotiana benthamiana*	202-1159	22,3-128,2	4,9-9,4	Zhong et al. (2023)
*Pisum sativum*	623-754	70.38-83.26	5.14-8.93	Li et al. (2023)
*Rosa chinensis*	369-891	N/A	N/A	Liu et al. (2021)
*Saccharum spontaneum*	441-1710	47,39-126,53	N/A	Wang et al. (2023)
*Sorghum bicolor*				
*Sesame indicum*	504-787	57.3-86.62	5.37-8.83	Yan et al. (2023)
*Solanum lycopersicum*	302-663	42.9-74.9	5.76-8.85	Sun et al. (2020)
*Solanum lycopersicum*	732-799	81.21-88.30	6.13-8.39	Kurt et al. (2020)
*Solanum tuberosum*	534-1045	59,98-116,77	5,20-8,33	Yu et al. (2022)
*Triticum aestivum*	N/A	65,4-119,2	5,09-9,24	Xia et al. (2022)
*Zea mays*	342-1328	38.03-147.35	4.96-8.96	Hu et al. (2023)

N/A means that that particular data was not discussed in the relevant publication.

Molecular confirmation of *in silico* predictions was achieved in cotton and barrel medic using GFP markers to demonstrate the subcellular localization of specific WAKs (Kong et al., 2023; Zhang et al., 2021). Five bread wheat *TaWAKs* fused with a *GFP* gene were transiently expressed in wheat protoplasts. Four of the five showed fluorescence only at the plasma membrane, while the GFP-TraesCS3D02G046900 showed fluorescence throughout the protoplast, with higher intensity at the plasma membrane and the nucleus (Xia et al., 2022). The authors suggested that the plasma membrane localization allows these proteins, in part, to act as receptors during immune responses, and the localization of one WAK to the nucleus warrants further investigation. These findings imply that TraesCS3D02G046900 functions by detecting DAMPs in the cytoplasm rather than the extracellular space, thereby signaling successful pathogen penetration that influences the expression of a different repertoire of defense-related genes compared to other WAKs. This particular protein sequence (TraesCS3D02G046900.1), identified as a WAK, contains the pkinase, GUB_WAK_bind, SP and TM domains but lacks an EGF domain, classifying it as a WAKL by earlier definitions (**Figure 2**). The bread wheat online annotation reveals eight different ORFs for this gene with some producing proteins lacking the TM domain (http://plants.ensembl.org/Triticum_aestivum/Gene/Summary?g=TraesCS3D02G046900) which could explain the dispersal of this protein throughout the cell. This emphasizes the need for a consistent definition of WAKs and WAKLs to improve comparability across this gene family to enhance our understanding of their function within the plant cell. Subcellular localization predictions provide a foundation for further molecular confirmation, because knowing where proteins function can inform their roles and downstream processes and the cell structures they influence.

### 6.2 Phylogenetic analyses for functional inference and conserved motif analyses

Phylogenetic analyses of newly identified WAK/WAKL protein sequences are constructed to assess the evolutionary relationships and to infer functions based on similarities to other known protein sequences (Sjölander, 2004). These phylogenetic analyses include the species-specific protein sequences or include previously characterised protein sequences from other species. The WAK/WAKLs typically form three to six phylogenetic clusters depending on the study. Despite using protein sequences, these clusters often reflect different gene structures, such as the number and placement of exons within the ORF. This has been observed in *N. benthamiana*, potato, tomato, and cannabis (Sipahi et al., 2022; Sun et al., 2020; Yu et al., 2022; Zhong et al., 2023). Phylogenetic clustering of proteins with similar gene structures provides an additional level of evidence for the role of tandem duplications within species, supporting the idea of gene family expansions within these species. In sorghum, however, the number of exons varies within groups, which suggests a different evolutionary process for the sorghum *WAK/WAKL* genes (Wang et al., 2023). Moreover, genes from different species with similar structures are often more closely related to each other than to those genes with differing structures within the same species. Species-specific clades in the *WAK/WAKL* gene family of walnut and cotton limit the ability to make broader functional inferences (Li et al., 2022; Zhang et al., 2021). The *Arabidopsis WAKs* are well studied, but often form independent clusters in phylogenetic analyses, hampering cross-species comparisons. This suggests that the *WAK/WAKL* gene family expands and evolves independently within each species, becoming more distinct over time (Zhang et al., 2021).

The composition of sequences used for phylogenetic analyses varies between studies. Some only include the species’ identified WAK/WAKLs, others incorporate *A. thaliana* sequences (including studies done in bread wheat and tomato) and some (potato and cannabis) include functionally characterised WAK/WAKLs involved in development, cell elongation and defense from various species such as *A. thaliana*, rice, cotton, maize, tomato, and apple (Sipahi et al., 2022; Sun et al., 2020; Xia et al., 2022; Yu et al., 2022). These analyses serve different purposes but often provide varying levels of characterization. A recent detailed phylogenetic analysis of 1061 *WAK* genes from 37 species revealed five clades, providing evidence of lineage-specific expansion after speciation, consistent with smaller-scale analyses (Zhang et al., 2023). The main purpose of these analyses is to assess relationships between previously characterised sequences, infer function, or predict the evolution of the gene family within the species. Including as many functionally characterised WAK/WAKLs as possible maximizes the potential for inferring function, especially since *A. thaliana* sequences often cluster independently, as seen in sesame, walnut and cotton WAK/WAKLs (Li et al., 2022; Yan et al., 2023). If the *WAK/WAKLs* of only the species of interest are included, inferences could be made on the evolution and expansion of the gene family within the species, allowing for a hypothesized description of the gene family’s history. Consistency in the approach would enhance comparability among the gene family as similar levels of description will be available.

When assessing conserved protein motifs, most characterizations use the online, open-source platform MEME (Multiple EM for Motif Elicitation - https://meme-suite.org/meme/tools/meme) (Bailey et al., 2015). MEME identifies near-exact repeating patterns of sequences (conserved motifs) with statistical modelling. The maximum number of conserved motifs is manually set, representing the best statistical set. To ensure comparability, the number of conserved motifs should be constant, ideally at 10, so that only the 10 most significant motifs can be compared between WAK/WAKLs from different species. Only cannabis, tomato and barley studies provided motif sequences, showing little similarity in motif compositions (Sipahi et al., 2022; Sun et al., 2020; Tripathi et al., 2021).

Ten motifs were identified in cannabis, potato, tomato, barley and *Saccharum* species, 12 in cotton, and 15 in *N. benthamiana* (Sipahi et al., 2022; Sun et al., 2020; Tripathi et al., 2021; Wang et al., 2023; Yu et al., 2022; Zhang et al., 2021; Zhong et al., 2023). Groupings obtained by phylogenetic analysis often align with motifs as seen in the group II cluster of potato WAKLs (Yu et al., 2022). *N. benthamiana* and *Saccharum* spp. WAK/WAKLs contain motifs specific to phylogenetic groups, with higher similarity within groups than those between groups (Wang et al., 2023; Zhong et al., 2023). The distribution and types of motifs in WAK/WAKLs are largely conserved within phylogenetic groups likely due to the absence of exon -gain and -loss over long evolutionary periods, indicating functional conservation (Sun et al., 2020; Yu et al., 2022; Zhong et al., 2023). In cotton, variations were mainly observed in the N-terminal of the proteins, where the SP, GUB_WAK_bind and EGF-Like domains are located (Zhang et al., 2021). Conserved domains were primarily in the C-terminal (STK domain), suggesting a highly conserved kinase domain for downstream functioning. Less conservation in the SP, the pectin binding and EGF-like domains could allow differential translocation between proteins, recognition of different pectin forms, and interaction with a wide range of additional proteins, respectively. Standardizing the number of predicted motifs at 10 will allow more direct comparisons across species, ensuring the best statistical set of representative motifs.

### 6.3 Impact of polymorphism on function

Polymorphisms in the WAK sequences can significantly affect protein functioning. The *RFO1* gene (annotated as *AtWAKL22*), is a key defense gene in *A. thaliana*, providing resistance against *Fusarium oxysporum* infection and was later described to detect changes in methylation status of cell wall pectin following infection (Huerta et al., 2023). Polymorphisms between ecotypes of this gene influence the protein’s efficiency in the defense response (Diener and Ausubel, 2005). The authors identified 21 polymorphisms between two ecotypes, including two 3 bp deletions as well as 10 causing missense mutations. One mutation changed the highly conserved glutamic acid (nucleotide 1652) in the resistant ecotype to glutamine in the susceptible ecotype. Similar sequence differences were seen in the *WAK2* genes of resistant and susceptible wheat, including a deletion, an insertion, and an A-to-C substitution (Gadaleta et al., 2019). Three independent *wak2* mutant wheat lines, with predicted loss-of-function mutations, exhibited severe disease symptoms. The authors hypothesized that these polymorphisms, along with alternative splicing, form different WAK protein variants, influencing the efficiency of the defense response. Focusing on the effect of polymorphisms within WAK/WAKLs among members of a species adapted to different biotic and abiotic stresses is important. Identifying these polymorphisms could improve *in silico* characterizations as an additional predictive tool for functional inferences. However, creating a comprehensive database of all known polymorphic regions with associated functionalities is essential before this type of *in silico* analysis can be implemented.

## 7 Discussion

**Figure 5:**
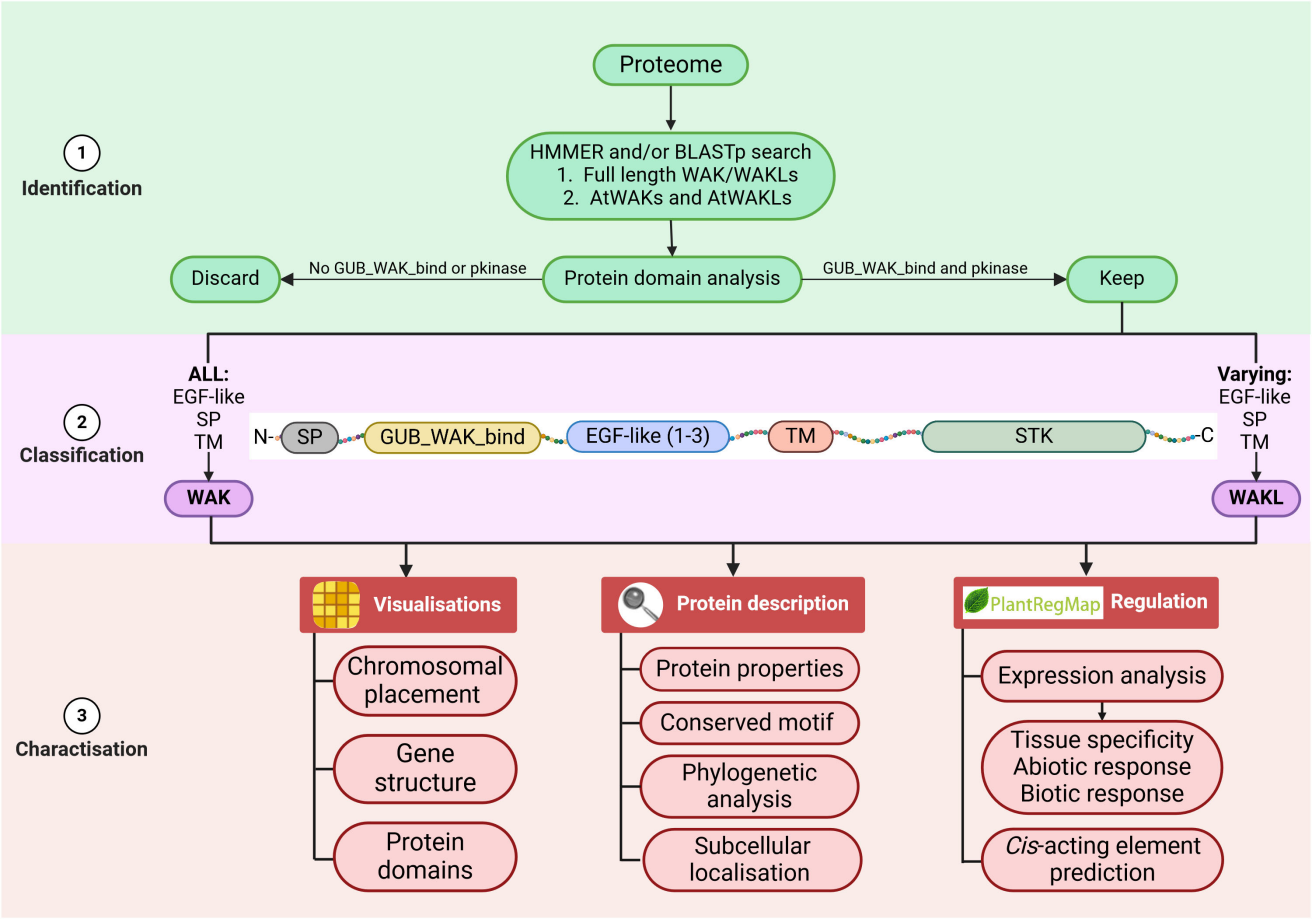
Proposed methodology for identifying, classifying and characterizing the *WAK/WAKL* gene family using species-specific proteomes. The outline specifies the identification methods, the minimum inputs required for classification, and which sequences should be excluded. Following classification, it lists the general analyses to be performed for a comprehensive *in silico* characterization of the gene family. (Created with BioRender.com).

The *WAK/WAKL* gene family has attained significant interest recently, leading to a substantial influx of foundational characterization studies. However, inconsistencies in the methodologies for identification, classification, and characterization of this gene family have hampered the comparability and robustness of the results. **Figure 5** provides a guideline to standardize the identification, classification, and characterization of *WAK/WAKL* gene families, aiming to streamline the process and improve consistency across different species.

The identification process begins with the predicted proteome of the species of interest. This proteome is used in a HMMER or BLASTp search utilizing full-length WAK/WAKL sequences, with at least the AtWAK/WAKL sequence, as the query. Using full-length proteins as inputs is crucial since domain-based HMMER searches may overlook genes lacking specific queried domains, given the previous variations in WAKL definitions. Only proteins identified with an e-value < 1e^-5^ should be considered for further investigation, as this is the typical threshold used. Proteins not containing both the GUB_WAK_bind and pkinase domains (or have the domains but with an associated e-value > 1e^-5^) should be discarded, while those that contain both domains are kept for classification. The classification of WAKs and WAKLs should follow the model proposed in this review, with all domains having an e-value < 1e^-5^ (**Figure 2**). Once classified, the *WAK/WAKL* genes can undergo thorough characterization to fully describe the family. This characterization provides information on the genes, their protein products, and their regulation for functional implication. Visualizations can be created using TBtools with information extracted from general feature format (gff or gff3) files for gene structure and chromosomal placement, and output from the NCBI CDD search for protein domains (Chen et al., 2020; Marchler-Bauer et al., 2011). Protein descriptions can be done by extracting the protein sequences and using tools such as Genefinity (http://www.geneinfinity.org/) for protein property predictions, MEME (https://meme-suite.org) for conserved motif prediction, Genious Prime or MEGA for the phylogenetic analysis using protein sequences and WoLF PSORT (https://wolfpsort.hgc.jp/) for subcellular localization prediction (Bailey et al., 2006; Horton et al., 2007; Tamura et al., 2021). Regulation can be assessed by visualizing expression data generated (or obtained from an online database) with R-studio or TBtools, while *cis*-acting elements can be predicted with PlantRegMap (https://plantregmap.gao-lab.org/) and/or PlantCARE (https://bioinformatics.psb.ugent.be/webtools/plantcare/html/) with *A. thaliana* as the reference species (Chen et al., 2020; Lescot et al., 2002; Racine, 2012; Tian et al., 2019).

By implementing this model for future characterizations, there will be an increased consistency across the *WAK/WAKL* gene family, facilitating better comparisons between and across species. To address previously described *WAK/WAKL*, the coding sequences should be made available for future researchers to confirm or adjust annotations using these guidelines for more accurate large-scale analyses. Both WAKs and WAKLs have been shown to be involved in various developmental and defense-related pathways, playing important roles in plants. Incorrectly identifying a WAKL as a WAK, or identifying a WAKL when it does not meet the minimum criteria, can impact future functional work looking into their biological significance, as there may be differences in modes of functioning due to differing defining features such as functional protein domains. Improved characterization and comparisons of gene families will enhance bioinformatic predictions based on previous data, leading to a better selection of candidate genes for functional analysis.
